# Detection of Friction Stir Welding Defects of AA1060 Aluminum Alloy Using Specific Damping Capacity

**DOI:** 10.3390/ma11122437

**Published:** 2018-11-30

**Authors:** Waheed Sami AbuShanab, Essam B. Moustafa

**Affiliations:** 1 Marine Engineering Department, Faculty of Maritime Studies and Marine Engineering, King Abdulaziz University, Jeddah 21589, Saudi Arabia; 2 Mechanical Engineering Departments, Faculty of Engineering, King Abdulaziz University, Jeddah 21589, Saudi Arabia

**Keywords:** friction stir welding (FSW), defects, detection, damping, capacity, dynamic, vibration

## Abstract

The demand for nondestructive testing has increased, especially in welding testing. In the current study, AA1060 aluminum plates were jointed using the friction stir welding (FSW) process. The fabricated joints were subjected to free vibration impact testing in order to investigate the dynamic properties of the welded joint. Damping capacity and dynamic modulus were used in the new prediction method to detect FSW defects. The data acquired were processed and analyzed using a dynamic pulse analyzer lab shop and ME’Scope’s post-processing software, respectively. A finite element analysis using ANSYS software was conducted on different types of designed defects to predict the natural frequency. The results revealed that defective welded joints significantly affect the specific damping capacity. As the damping ratio increased, so did the indication of opportunities to increase the presence of defects. The finite element simulation model was consistent with experimental work. It was therefore revealed that natural frequency was insufficient to predict smaller defects.

## 1. Introduction

Friction stir welding (FSW) and processing is an advanced technique used for the joining and fabrication of aluminum alloys. As a result of different processing parameters, such as welding speed and tool shape, discontinuities occur in welded joints, including channel cavities and porosities. Many researchers have studied weld defects and their classification [1,2,3,4,5,6,7,8]. Welding speed is one of the most important processing parameters during the FSW process, and the heat input generated has been reported to have a significant influence on the welding process [9,10,11,12,13]. The quality of FSW depends on the speed of the rotating welding tool as the source of input heat in the process. The formation of tunnels, wormholes, and voids in the welded joint is caused by insufficient heat input and deficiencies in the material flow [14,15,16].

Internal defects are an important type of defect that are difficult to examine using traditional methods [17]. Traditional nondestructive test methods, such as ultrasound, radiography, and eddy current, are used to detect welding defects but they cannot test and inspect all types of materials. Some of these methods are limited to the detection of ferrous metals and the measurement sensors are sensitive to imperfections in the welded surface [18,19,20]. An automatic identification algorithm was investigated to detect and classify weld defects using radiographic images [21]. Many studies used vibration and acoustic emissions to identify the dynamic characteristic behavior of materials to predict defects [22,23,24,25]. The power spectrum density for the frequency component was used as a good indicator of welding defects [26]. Online monitoring of the welding process and defects was investigated by Chen et al. [27], using wavelet transform of the acoustic emission signal during friction stir welding to detect the welding defects and weld state. A fractal dimension algorithm was used to extract different acquired signals in order to monitor the welding process and detect the defects due to changes in the signal values [28]. Thermography was used as an online monitoring technique in order to evaluate the quality of the welded joints using the FSW process by monitoring the thermal profile during the welding process [29,30].

A free vibration method was used as a nondestructive test to determine the quality of the welding [31], which depended on the natural frequencies of the welded samples. The authors concluded that higher modes of natural frequencies better indicated the surface welding defects.

Most of the previous investigations concerning the dynamic properties of welding focused on natural frequency, and very small defects were not detected. In the present work, damping capacity is used to more accurately detect and identify FSW defects using free vibration analysis. The new detection method depends on the variance in the values of the damping ratio and other dynamic properties. The results are verified using radiographic analysis. Finite element analysis using ANSYS software was carried out to simulate and predict the natural frequency of the defective joint. Through this study, we show that natural frequency alone is insufficient for detecting welding defects.

## 2. Experimental Procedure

**Figure 1:**
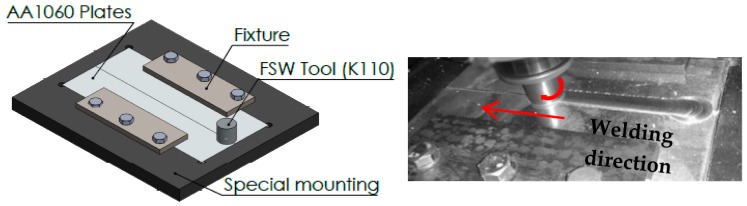
Experimental friction stir welding (FSW) process of AA1060 aluminum alloy.

**Figure 2:**
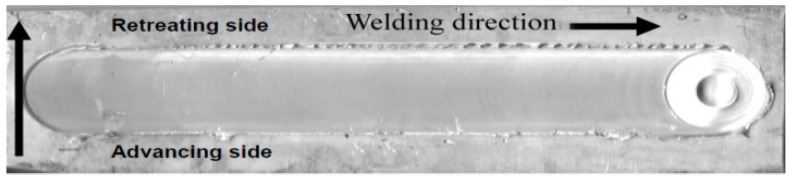
Welded plates after FSW process.

Aluminum alloy AA1060 plates were used as the base metal. The chemical composition of this commercial alloy is presented in Table 1. It is widely used in special tanks and chemical industries on account of its corrosion resistance. Moreover, as it has high thermal and electrical conductivity, it is commonly used in electrical applications. The specimens were cut into rectangular shapes 250 mm long, 60 mm wide, and 6 mm thick. A high carbon-chrome steel (K110) tool with a shoulder diameter of 25 mm, pin diameter of 6 mm, and length of 5 mm, was used for the welding process. The process was performed at different rotation and traverse speeds, with five tool rotation speeds being chosen (600, 1000, 1200, 1500, and 1800 rpm). Each rotation speed was applied with four different welding speeds (16, 32, 52, and 110 mm/min). The tool was used with a tilt angle of 2 degrees. An automatic milling machine (Bridgeport, Elmira, NY, USA) was used to perform the friction stir welding process, as shown in Figure 1. After performing the welding process, the samples were slightly machined on the surface of the welded joint in order to remove the flashes and other irregularities generated during the FSW process, taking care to not remove any surface defects. Most of the generated defects formed inside the welded joint and did not appear visually in most of the welded samples, as shown in Figure 2. After that, we cut the joints longitudinally in the middle of the welding position to create 21 mm wide specimens. The specimens (200 mm× 21 × mm × 4 mm) were prepared for the vibration test and radiography inspection. The samples were polished and etched in the cross-section of the friction stir welding joint, using classic Keller’s reagent (2 mL HF (48%) + 5 mL HNO_3_ +3 mL HCL + 190 mL distilled water) for the macroscopic examination.

### Free Vibration Impact Test

**Figure 3:**
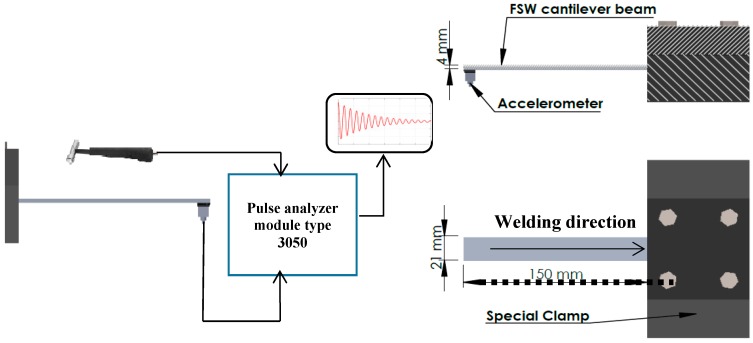
Vibration apparatus and free vibration test setup.

Samples were prepared as a cantilever beam with one free end and fixed using a special clamp. The net fixed dimensions of the tested cantilever beam were 150 mm long, 21 mm wide, and 4 mm thick. The vibration signal was acquired using a piezoelectric CCLD accelerometer. The accelerometer was mounted using plastic clips. An impact hammer with a force transducer was used to excite the welded joint. Vibration analysis was performed using a LAN-XI (3050 A-60 Bruel&Kjaer, Nærum, Denmark). The data acquisition system was a 6-channel input module, with a frequency range of (0–51 KHz), FFT resolution up to 6400 lines. Figure 3 shows the apparatus used in the investigation. The specific damping ratio, natural frequency, and frequency response function were analyzed using post-processing software ME’Scope (Vibrant Technology, Centennial, CO, USA) and verified using theoretical methods. The test was repeated 10 times for each sample in order to attain the optimum value through average readings.

## 3. Results and Discussions

The main purpose of this investigation was to predict FSW defects using the dynamic properties of a welded joint. The vibration analysis was calculated and analyzed using commercial vibration analysis software (Pulse Labshop and ME’Scope). Empirical and theoretical equations were used to verify the estimated dynamic properties, such as the damping ratio and natural fundamental frequency. The damping capacity refers to the ability of a material to absorb energy. Materials with a high damping capacity often indicate weakness in the material, and from this principle [32], it is possible to predict the welding defects.

### 3.1. Free Vibration Analysis

In this method, which is also called the damped free vibration method, the specimen freely vibrates at its natural frequency, and is damped only by the internal friction of the material. The amplitudes of successive cycles are determined and used to calculate the logarithmic decrease according to Equation (1). The logarithmic decrease is one of the most common methods used to express the damping capacity, as presented in Equations (1)–(3). The logarithmic decrease represents the rate at which the amplitude of a free damped vibration decreases [33,34,35]. Thus, the logarithmic decrease δ is obtained as:

(1) $$\delta =\frac{1}{n}\ln\frac{x_{o}}{x_{n}}$$

(2) $$\zeta =\sqrt{\frac{\delta^{2}}{\delta^{2}+4\pi^{2}}}$$

where x_o_, x_n_, and n are the amplitudes of the first and last cycles, and number of cycles, respectively. The damping ratio is calculated using Equation (2), where ζ is the damping ratio.

Specific damping capacity (ψ) = 2δ
(3)

**Figure 4:**
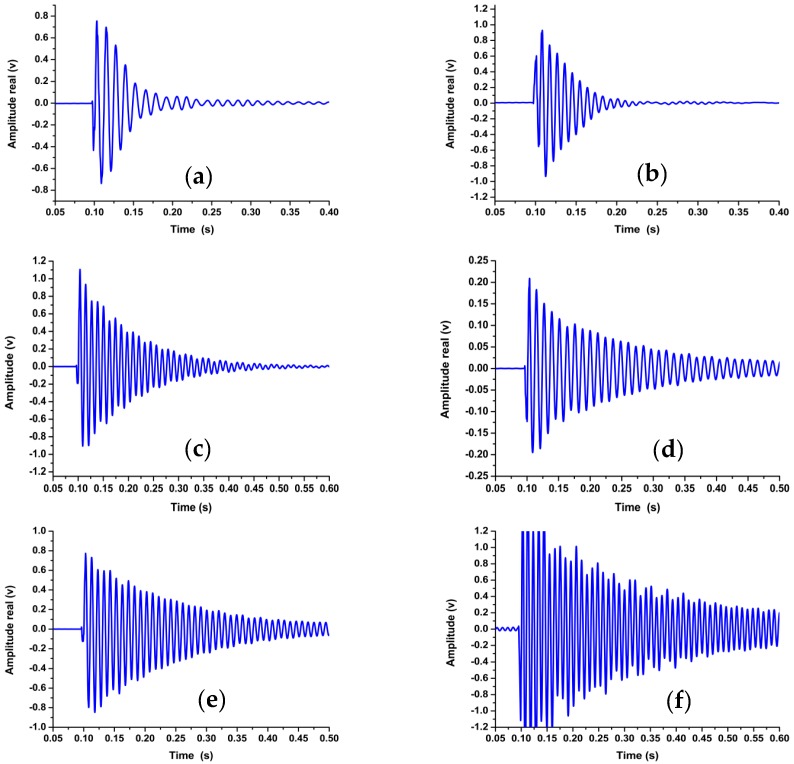
Transient response acquired from the free vibration impact test at (**a**) 600 rpm with a welding rate of 16 mm/min, (**b**) 1500 rpm with a welding rate of 52 mm/min, (**c**) 1500 rpm with a welding rate of 110 mm/min, (**d**) 600 rpm with a welding rate of 110 mm/min, (**e**) 1200 rpm with a welding rate of 32 mm/min, and (**f**) 1000 rpm with a welding rate of 110 mm/min.

Figure 4 shows the different decay curves for the defective and defect-free samples, as noted in the decay curves (Figure 4a,b).

The time required to dampen the vibration was minimal when compared to other cases (Figure 4e,f). Therefore, the defective welded joints impede the pulse signal by damping its velocity and amplitude. For defect-free samples, the transmitted signal decays normally and increases the time required to be steady in the transient response domain. Defective welded samples require approximately one-third of the time required by defect-free welds to be damped.

### 3.2. Effect of Process Parameters on Damping Capacity

**Figure 5:**
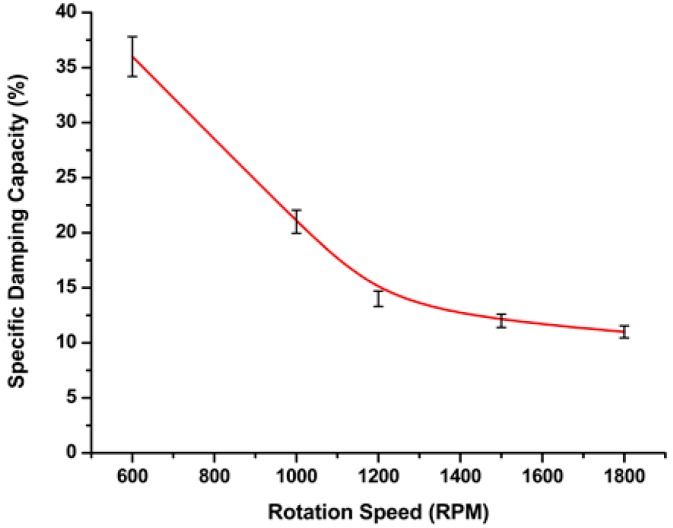
Reciprocal fit of specific damping ratio and tool rotation speed at a constant welding speed of 32 mm/min.

The effect of FSW processing parameters, such as the welding speed rate and tool rotation speed, on the formation of defects was observed using the variance in the damping capacities in the tested welds. The specific damping capacity and the FSW tool rotation speed (ω, rpm) were formulated using the regression analysis in Equation (4). As this represents the regression model, we formulated the relationship between the processing parameters (rotation speed) of friction stir welding and the corresponding damping ratio. At a lower rotation speed, the specific damping capacities have higher values than at high speeds, as shown in Figure 5.

(4) $$\mathrm{specific}\;\mathrm{damping}\;\mathrm{capacity}\;\left(\psi \right)=\frac{1}{a+\left(b\times \omega \right)}$$

Here, *a* and *b* are the constants of the regression equation, *a* = −0.00767, *b* = 5.89567 × 10^−5^; and ω is the rotation speed in rpm.

**Figure 6:**
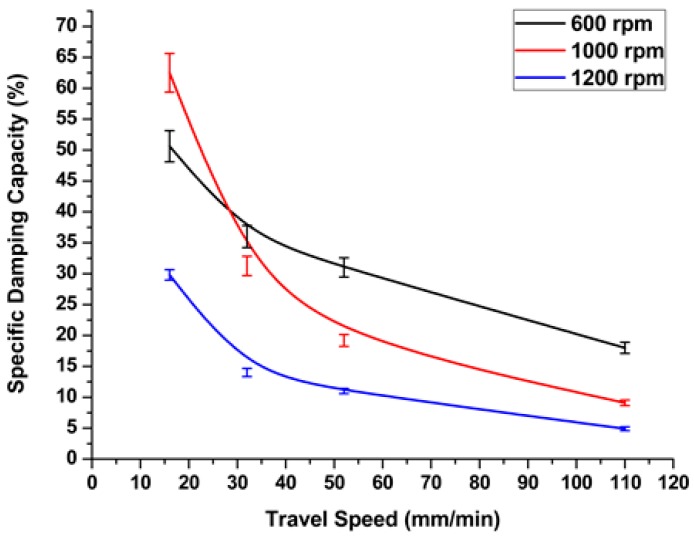
Effect of welding rate on damping capacity at different rotation speeds.

**Figure 7:**
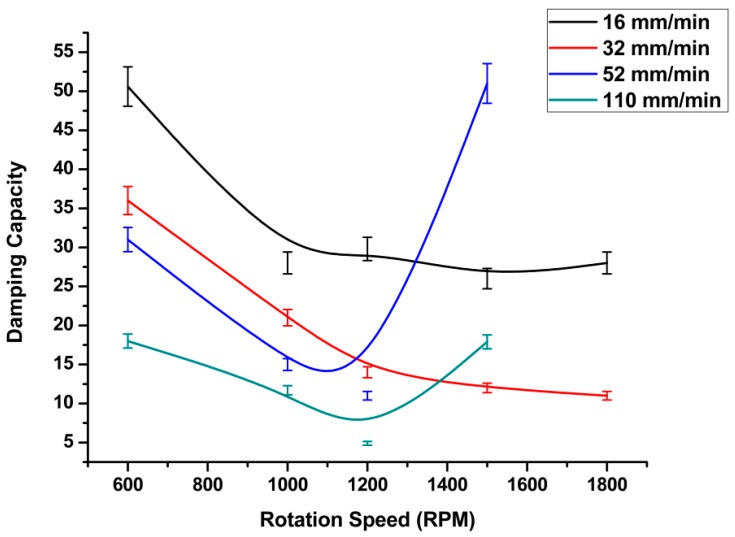
Effect of FSW processing parameters on damping capacity.

In addition to the rotation speed, one of the main processing parameters that affects the friction stir welding process is the traverse speed (welding rate) (*v*, mm/min). From the experimental results, we observed that whenever the welding rate increased, the heat generated during the friction stirring process decreased. The specific damping capacity decreased nonlinearly with respect to the welding speed, as shown in Figure 6. From the above, higher tool rotation speeds and the corresponding welding speeds caused an increase in damping capacity values, which were reflected in the quality of the welding process, causing defective welds. However, all welding speeds at low rotation speeds show a significant increase in damping values, as shown in Figure 7. Higher welding speeds, with a corresponding tool rotation speed of above 1100 rpm, showed a dramatic increase in damping ratio. This can be explained by the fact that a higher rotation speed, when processed at a high welding rate, causes insufficient heat in the stirring zone. Therefore, defects will form during the welding process and, consequently, the damping ratio increases. 

Table 2 presents the regression function parameters. Regression Equation (4) was used with minimal error, whereas the error represents the error of regression parameters (a and b), and thus expresses the fitting accuracy of the equation. At lower welding rates with a low tool rotation speed, we observed that the damping capacity increased, indicating that the total mass and the stiffness of the welded joint decreased. The time domain of the tested samples showed significant changes in the decay time for the defective and defect-free samples.

**Figure 8:**
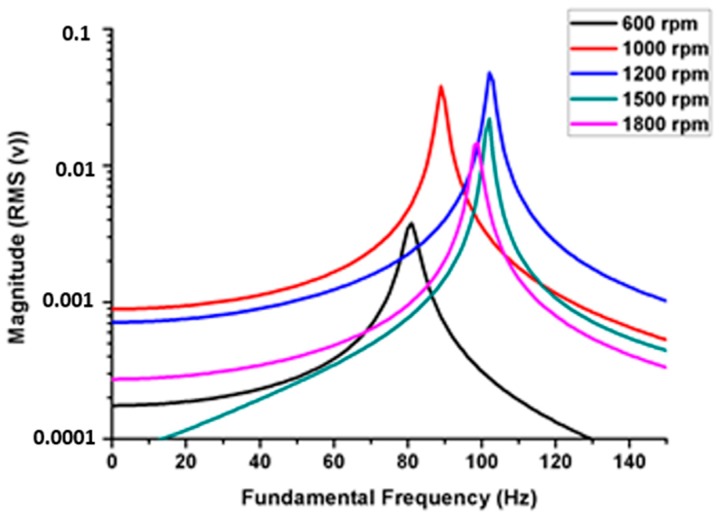
Frequency response function of different rotation speeds at a constant welding rate of 32 mm/min.

The natural frequency was affected by internal defects: as the welded joint stiffness decreased, the natural frequency decreased. The tunnel and generated voids were influenced by the natural frequency of the welded joint, and the internal defects acted as an absorber medium for the acoustic and vibration signals. At higher rotation speeds, we observed an increase in the natural frequency, as shown in Figure 8. However, the increase in natural frequency was not indicative of weld quality.

### 3.3. Effect of Pseudo Heat Index (ω^2^/ν) on Damping Capacity

**Figure 9:**
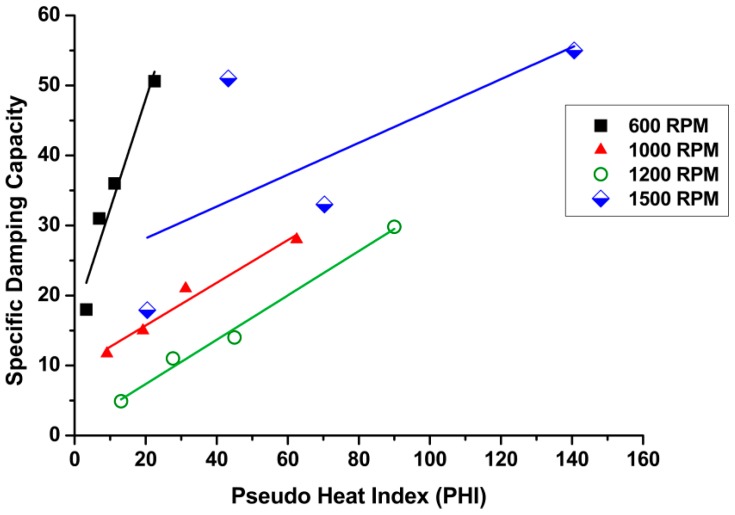
Effect of pseudo heat index (PHI) on damping capacity.

The ratio of the square of the tool rotation speed to linear welding speed is defined as the pseudo heat index (PHI). This method is used to predict the net heat generated during friction stir welding [36]. Figure 9 represents the relationship between the PHI and the specific damping capacity for all rotation speeds and corresponding traverse speeds. It can be seen that any significant increases in the heat index parameter led to increases in the damping capacity. Therefore, it was possible to predict the presence of defects in welding, especially when both parameters increased.

### 3.4. Effect of Welding Defects on Dynamic Properties

The dynamic modulus was calculated according to ASTM Standard E1875-08, using Equations (5) and (6) [37]. Table 3 presents the calculated values that focused on the major defective welded joints, whereas Table 4 presents the defect-free samples. From the calculations, we observed that the dynamic Young’s modulus decreased in the presence of defects.

(5) $$E_{d}=0.9465\;\left(\frac{mf^{2}}{b}\right)\left(\frac{l^{3}}{t^{3}}\right)T$$

where *E_d_* is the dynamic modulus, *m* is the mass of the bar, *b* is the width of the bar, *L* is the length of the bar, *t* is the thickness of the bar, *f* is the natural frequency of the bar in flexure, and *T* is the correction factor for fundamental flexural mode to account for the finite thickness of the bar.

(6) T=1.000+6.585(t/l)

The dynamic modulus was calculated using experimental results from the vibration analysis and the measurement of the sample mass and volume in order to obtain the density. According to ASTM Standard E1875-08 [37], errors occur in experimental calculations due to measurement errors; therefore, these errors were approximately in the range of ±1.8%.

The dynamic properties of the welded joint reflect the quality of the welds through three main factors: natural frequency, damping capacity, and dynamic modulus. The dynamic modulus depends on the natural frequency, mass, and volume of the welded sample. The effect of natural frequency cannot accurately detect defects in welding joints, and this is explained in the next section through the simulation model to predict the natural frequency of defective welds. The second parameter is the mass of the sample, where internal defects decrease the mass. Hence, the defect forms an empty cavity within the metal or welds. When the mass decreases in addition to the slight decrease in the natural frequency, the dynamic modulus also decreases. The experimental results revealed that lower dynamic modulus values were observed at higher damping capacity values.

### 3.5. Verification Tests

#### 3.5.1. Radiography Scan

**Figure 10:**
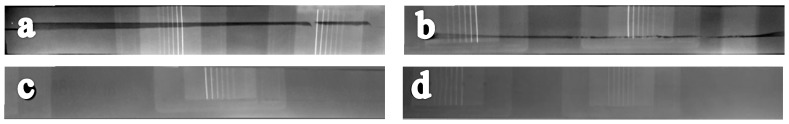
Radiography scan at (**a**) 600 rpm with a welding rate of 16 mm/min, (**b**) 1500 rpm with a welding rate of 52 mm/min, (**c**) 1200 rpm with a welding rate of 52 mm/min, and (**d**) 1200 rpm with a welding rate of 32 mm/min.

In this test, the welded joints were subjected to a nondestructive radiography scan (YMGI, YXLON, Hudson, NY, USA), to inspect the internal defects of all the samples. The test was performed according to E 1032–95, the Standard Test Method for Radiographic Examination of Weldments. The main purpose of this test was to verify the current investigation using a traditional nondestructive test. The radiography scan uses an X-ray beam projected onto the welded joints. The depth of the defect in the direction of the radiation beam was obtained from the density profile of the defect. As shown in Figure 10, the majority of the defective joints had a tunnel defect, as shown in Figure 10a,b. The results were consistent with the dynamic properties and specific damping capacity. In contrast, the radiography scan did not show any variation in the density profile for defect-free samples, as shown in Figure 10c,d.

#### 3.5.2. Macrograph Inspection

**Figure 11:**
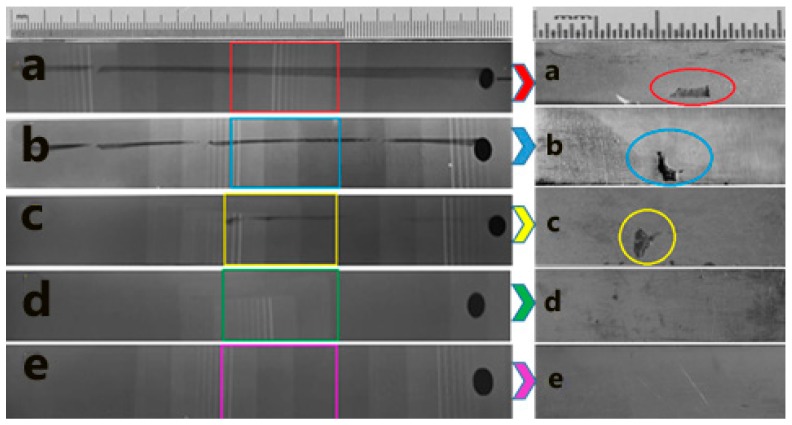
Radiography scan with corresponding section using macro-scan: (**a**): 600 rpm with a welding rate of 16 mm/min, (**b**) 1500 rpm with a welding rate of 52 mm/min, (**c**) 1800 rpm with a welding rate of 32 mm/min, (**d**) 1200 rpm with a welding rate of 32 mm/min, and (**e**) 1000 rpm with a welding rate of 110 mm/min.

The welded samples were verified using a destructive sectioning method after determining the position of the cavities and defects in each sample using previous nondestructive radiography scans. Figure 11 shows the macrographs and the section position for each sample. The macrograph was carried out by cutting the welded joint according to the radiograph scan to show the defect profile in each defective sample. The macrograph pictures revealed that most of the defective samples were away from the surface. Furthermore, the defects tended to occur down surface from the FSW joint. The shape of the tunnels and cavities helped to simulate the defects in the next section in the finite element analysis. These shapes were irregular as a result of the processing parameters, tool shape, and material type.

### 3.6. Finite Element Analysis

**Figure 12:**
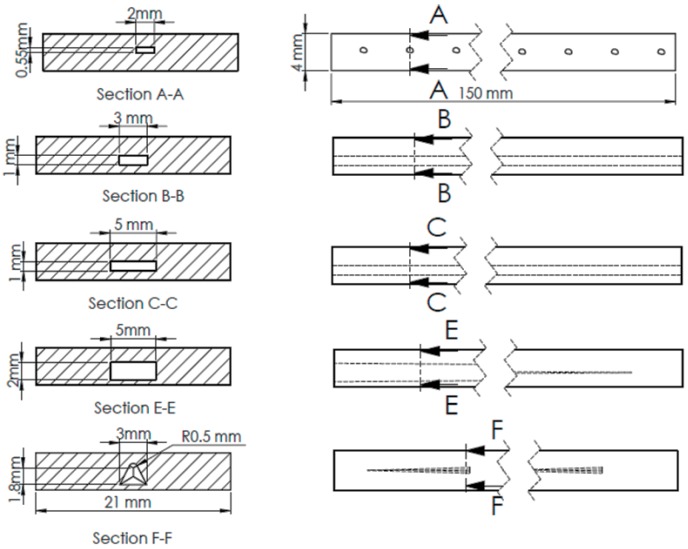
Different designs of FSW defects used in finite element analysis (FEA) simulation. (**A**) linear porous defect, (**B**) regular tunnel type B, (**C**) regular tunnel type C, (**E**) taper rectangle tunnel, and (**F**) intermittent taper triangle tunnel.

Finite element analysis (FEA) was carried out using modal analysis and harmonic response modules. Different models of welding defects were designed in order to simulate the mode shapes of the models and calculate the first natural frequency of each design. The welded joint was modeled as a cantilever beam with the same dimensions as the experimentally tested samples using the free vibration impact test discussed previously. Figure 12 shows the different designs of defects based on the most common defect results in the friction stir welding process.

The defect shape was modeled to the nearest regular form for analysis purposes. Hence, the difference between the regular shape and the actual irregular shape (from the macrograph) was very close in the final results. The simulation process involved two steps: loading the modal analysis; and designing suitable parameters to calculate the natural frequency. The meshing type and sizing were adjusted based on tetrahedron methods in order to perform the simulation with minimal error. Furthermore, boundary conditions were applied to the end of a cantilever beam as a fixed support, and the force was provided at the tip of the beam. The second step (harmonic response) was conducted as a simulation process in order to predict the frequency response function (FRF).

**Figure 13:**
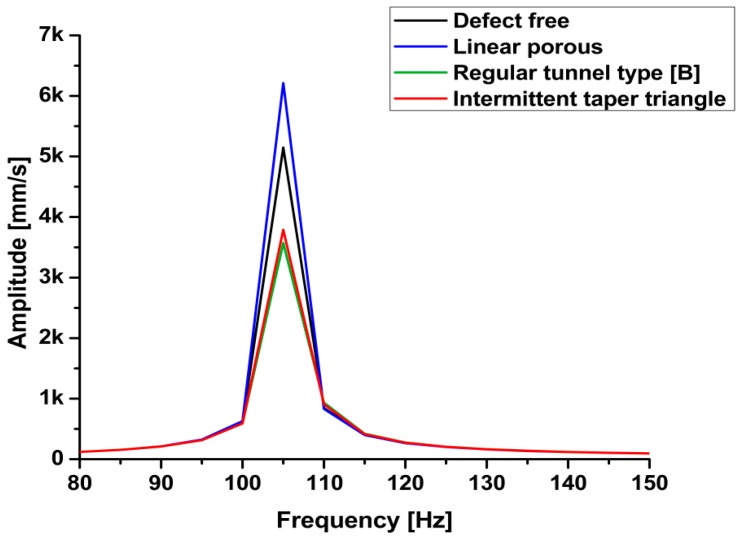
Frequency response function (FRF) simulation of small defects.

**Figure 14:**
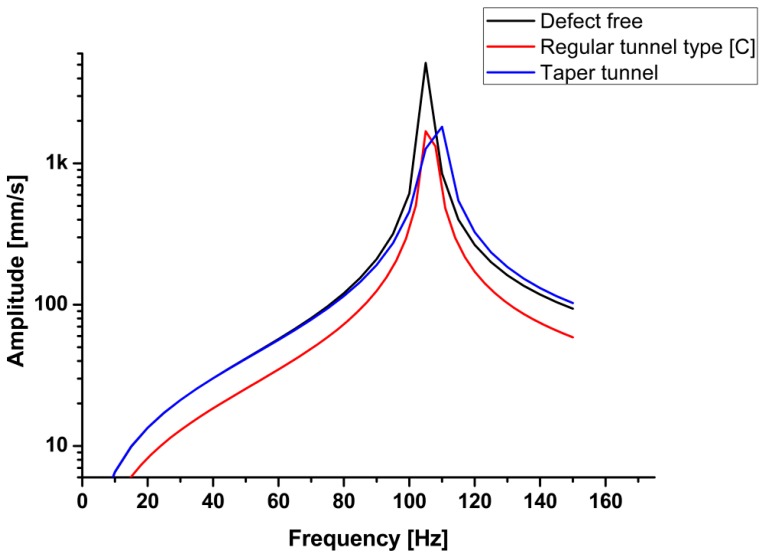
FRF simulation of large tunnel defects.

All the calculations and essential data were collected based on the previous processing step. The frequency range was adjusted to 0 to 150 Hz according to the natural frequency calculation in the first step. Six models were built and simulated using finite element software (ANSYS, ANSYS Inc, Canonsburg, PA, USA). In the present investigation there are six types of welding defect have been simulated using finite element analysis software Each model represented a welded joint under certain defects, except the first one, which was defect-free. The models simulate some of the common experimental defects generated during welding process. The models were designed to form defects varying from the smallest to the largest in size. Linear porous defect in section A-A represents the smaller defect. The other models represent: regular tunnel type section B-B, regular tunnel type section C-C, taper rectangle tunnel section E-E, and intermittent taper triangle tunnel section F-F. Table 5 presents the results of the simulation of different designs of defects. The results revealed that the values of the natural frequency of the welded joint were very close, whether defective or not, because the size of the defect does not significantly affect the mass, which impacts the natural frequency. Close natural values were observed in small defects with the defect-free sample. Small defects, such as pores and small cracks, cannot be detected through the natural frequency of a metal because the variation in frequency is limited, as shown in Figure 13. Those defects significantly affected the welded structure. In the case of noticeable defects, the natural frequency can detect these defects, although the values of the defective sample frequencies converge with those of the defect-free sample, as shown in Figure 14. Table 6 presents a comparison of the results between the experimental and simulated natural frequency for the defect-free samples and for significant defects. The simulated results show that the natural frequency increases slightly with small detects, contrary to the experimental results, where the natural frequency tends to decrease as the defects increase.

The shape of a defect affects the stiffness of the welded joints. When the sample mass decreases, the natural frequency does not decrease at the same rate. From the previous figures, natural frequency is not a factor that can be used to detect welding defects. The results of the finite element analysis were consistent with the experimental vibration test, from which the dynamic properties could be deduced.

## 4. Conclusions

From previous results, we concluded that the FSW processing parameters affect the dynamic properties of the welded joint, according to the heat generated due to the stirring action and different processing conditions. The processing parameters that influence the dynamic characteristics are natural frequency, damping capacity, and dynamic modulus.

Specific damping capacity decreased nonlinearly with increases in the tool rotation speed and welding rate. This parameter expresses the welding quality because the damping capacity depends on the internal friction of the material. Consequently, specific damping capacity is an excellent parameter to detect welding defects.

The corresponding natural frequencies of the defective welding samples increased slightly with respect to the defect-free weld. Therefore, the experimental results of the FRF agree with the numerical method using the FEM results to show that the natural frequency of the defective joint has no significant effect on the detection of internal defects, especially if these defects are small.

## Figures and Tables

**Table 1:** Chemical composition of AA1060 (wt. %).

Alloy	Si	Fe	Cu	Mn	Mg	V	others	Al
1060	0.25	0.4	0.05	0.05	0.05	0.05	0.03	remain

**Table 2:** Parameters of the reciprocal function.

Tool Speed (ω)	*a*	Error	*b*	Error
600	0.01392	0.00124	3.83432 × 10^−4^	4.47684 × 10^−5^
1200	0.00258	0.00495	0.00195	2.6868 × 10^−4^
1500	0.02993	0.02455	5.54028 × 10^−5^	4.45066 × 10^−4^

**Table 3:** The dynamic properties of defective welds.

Welding Speed (mm/min)	Rotation Speed (rpm)	PHI (ω^2^/ν × 1000) (%)	Natural Frequency (Hz)	Dynamic Young’s Modulus (GPa)	Specific Damping Capacity	Calculation Error (%)
16	600	22.5	78	51.8	50.6	±1.8
16	1500	3.3	87.8	53.2	55	±1.8
52	1500	43.3	87	58.3	51	±1.8

**Table 4:** The dynamic properties of the defect-free welds.

Welding Speed (mm/min)	Rotation Speed (rpm)	PHI (ω^2^/ν × 1000) (%)	Natural Frequency (Hz)	Dynamic Young’s Modulus (GPa)	Specific Damping Capacity	Calculation Error (%)
110	1000	9.09	103	73.1	11.7	±1.8
32	1200	45	102	74.5	14	±1.8
52	1200	27.7	96	70.9	11	±1.8

**Table 5:** Investigated defect types and properties with corresponding frequencies.

Defect shape	Sample Mass (g)	Percentage of Mass Loss (%)	Natural Frequency (Hz)
Defect-free	35.02	0	105.69
Linear pattern porous defect	34.96	0.17	105.58
Regular tunnel type B	33.65	3.91	106.02
Regular tunnel type C	32.82	6.28	106.31
Taper rectangle tunnel	33.18	5.25	107.92
Intermittent taper triangle tunnel	34.59	1.23	105.95

**Table 6:** Comparison between experimental and simulated frequency results.

Defect Type	Experimental F_n_	Simulated F_n_
Defect-free	103	105.69
Linear pattern porous defect	-	105.58
Regular tunnel type B	87	106.02
